# ATPase-dependent duplex nucleic acid unwinding by SARS-CoV-2 nsP13 relies on facile binding and translocation along single-stranded nucleic acid

**DOI:** 10.1016/j.jbc.2025.110373

**Published:** 2025-06-12

**Authors:** Jinwoo Park, Yong-Joo Jeong, Khushbu Chauhan, Hye Ran Koh, Dong-Eun Kim

**Affiliations:** 1 Department of Bioscience and Biotechnology, Konkuk University, Seoul, Republic of Korea; 2 School of Applied Chemistry, Kookmin University, Seoul, Republic of Korea; 3 Department of Chemistry, Chung-Ang University, Seoul, Republic of Korea

**Keywords:** ATP hydrolysis, duplex nucleic acid unwinding, nucleic acid binding affinity, SARS-CoV-2 nsP13 NTPase/helicase, slowly hydrolysable ATP analog

## Abstract

Nonstructural protein 13 (nsP13) of severe acute respiratory syndrome coronary virus (SARS-CoV-2) is a superfamily one helicase, which is essential for viral RNA replication. This protein can unwind dsRNA and DNA with a 5′ single-stranded tail in the 5′–3′ direction. Previous studies have demonstrated that nsP13 efficiently unwinds double-stranded nucleic acids with a single-stranded tail through a cooperative translocation fueled by ATP hydrolysis. However, the mechanism underlying the aforementioned unwinding remains unclear. In this study, we hypothesized that the differences in unwinding efficiency among duplex nucleic acids are driven by the ATP hydrolysis–induced changes in the binding affinity of nsP13 to a single-stranded tail. When nsP13 unwinds dsDNA with a 5′ single-stranded tail, a long 5′ single-stranded tail enhances ATP hydrolysis and promotes DNA unwinding efficiency. When the slowly hydrolyzable ATP analog adenosine-5′-*O*-3-thiotriphosphate was used for dsDNA unwinding by nsP13, duplex DNA unwinding was largely diminished, whereas the binding affinity to the single-stranded DNA was more enhanced compared with ATP. Thus, unhindered ATP hydrolysis may allow nsP13 to bind and translocate along the single-stranded nucleic acid, resulting in the efficient unwinding of duplex nucleic acids.

Severe acute respiratory syndrome (SARS) is a respiratory infection caused by a novel coronavirus (SARS-CoV-2), which resulted in a considerable number of fatalities in 2019 (1). SCV is an enveloped, positive-sense, single-stranded RNA virus ((+) ssRNA virus) with a genome size of approximately 30 kb (2, 3). The first two-thirds of the SARS-CoV-2 genome encodes the replicase gene orf1ab, which produces 16 nonstructural proteins (nsPs). The open reading frames (ORF1a and ORF1b) of these replicase genes are translated into two large polyproteins, namely, pp1ab (∼790 kDa) and pp1a (∼490 kDa), *via* a −1 ribosomal frameshift (4). Then, these polyproteins are processed by viral cysteine proteases, such as MPRO or 3CLPRO, to yield 16 nsPs, including RNA-dependent RNA polymerases (RdRp and nsP12) and an NTPase/helicase (nsP13) (5, 6, 7). These viral replicases form the core of membrane-bound replication–transcription complexes that synthesize a full-length viral genome and multiple subgenomic mRNAs (6, 7, 8, 9).

Considering that viral helicases play an important role in viral replication and propagation, they represent key targets for antiviral drug development (10, 11, 12, 13, 14, 15, 16, 17). Targeting these enzymes could inhibit viral metabolism with minimal adverse effects (18). Consequently, considerable effort has been invested in developing small-molecule inhibitors and chemical compounds to inhibit the SARS-CoV-2 nsP13 helicase (14, 15, 17). nsP13 serves as a motor protein, which unwinds ds(ds) nucleic acids into single strands using the energy released from NTP hydrolysis, thereby facilitating nucleic acid replication, recombination, and repair (7, 19, 20, 21, 22). SARS-CoV-2 nsP13 can unwind double-stranded RNA (dsRNA) and double-stranded DNA (dsDNA) with a 5′ single-stranded tail, exhibiting 5′–3′ polarity, and it can hydrolyze all types of deoxyribonucleotide and ribonucleotide triphosphates (5, 20, 23, 24, 25). While the primary physiological substrates of the SARS-CoV helicase nsP13 are RNA structures involved in viral replication, our previous studies have shown that nsP13 can unwind both RNA and DNA duplexes with a 5′ to 3′ polarity and that both ssRNA and ssDNA stimulate its ATPase activity to a similar extent (23, 26). This dual specificity highlights the helicase’s intrinsic mechanistic flexibility and justifies the use of DNA substrates for probing fundamental aspects of its activity.

Based on a previous report, the amount of unwound dsRNA was reduced when nsP13 helicase unwinds dsRNA substrates as compared with dsDNA substrates, whereas increasing ATP concentration during dsRNA unwinding by nsP13 enhanced the amount of unwound dsRNA (23). During dsRNA unwinding by nsP13, a long 5′-ssRNA tail in the dsRNA substrate reduces the amount of dsRNA unwound by nsP13 (23), which is contrary to the enhanced unwinding efficiency in dsDNA with a long 5′-ss tail (26). The reduced dsRNA unwinding efficiency was attributed to the increased binding of nsP13 onto the ssRNA tail when increasing amounts of nsP13 were applied to the dsRNA substrate with an ssRNA tail (23). These contradictory trends in duplex nucleic acid unwinding are due to the different binding affinities of nsP13 onto the single-stranded tail, which serves as a loading site for multiple nsP13 monomers. However, the detailed mechanism underlying the higher duplex nucleic acid unwinding efficiency of nsP13 with dsDNA containing a single-stranded DNA (ssDNA) tail, compared with that with dsRNA containing an ssRNA tail, remains unknown. As the increased ATP concentration relieves the diminished dsRNA unwinding efficiency, the relationship between ATP hydrolysis and the binding of nsP13 onto the single-stranded tail of the duplex nucleic acid substrate needs to be investigated.

In this study, a slowly hydrolyzable ATP analog, namely, adenosine-5′-*O*-3-thiotriphosphate (ATPγS), was used to investigate the relationship between ATP hydrolysis and the efficiency of dsDNA unwinding by nsP13. When partial duplex DNA was used as a substrate for nsp13 unwinding, the presence of ATPγS inhibited the unwinding of duplex DNA and the ATPase activity of nsP13. The decrease in dsDNA unwinding efficiency in the presence of ATPγS was examined to determine whether the binding affinity of nsP13 onto ssDNA increased in the case of hindered ATP hydrolysis. ATPγS strongly binds nsP13 onto the ssDNA tail but fails to allow nsP13 to translocate along the ssDNA track because of abolished ATP hydrolysis. Thus, we suggest that duplex nucleic acid unwinding by nsP13 relies on facile binding and translocation along single-stranded nucleic acids with uninterrupted ATP hydrolysis.

## Result

### Fluorometric detection of duplex nucleic acid unwinding by nsP13

**Figure 1:**
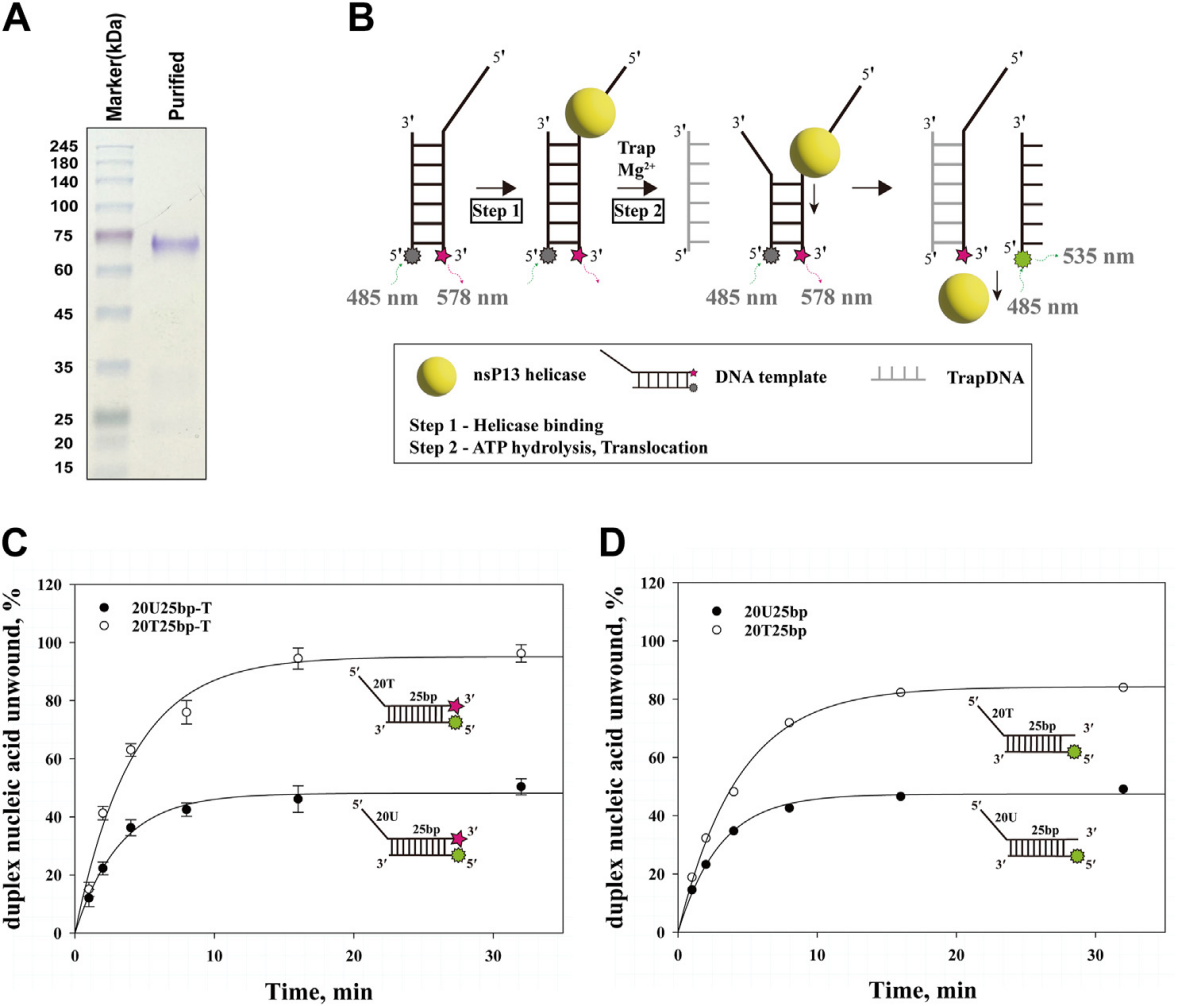
**Biochemical analysis of duplex nucleic acid unwinding by purified recombinant nsP13.***A*, SDS-PAGE analysis of purified recombinant nsP13 protein, visualized by *Coomassie Blue* staining. *B*, schematic of the FRET-based assay used to monitor nsP13 helicase activity on duplex nucleic acids. Unwinding of duplex nucleic acid substrate was carried out in the presence of a trap oligonucleotide (DNA or RNA) that corresponds to the *bottom* strand of the duplex nucleic acid substrate. *C*, FRET-based unwinding assay of RNA (•, 20U25bp-T) and DNA (○, 20T25bp-T) duplexes over time at 37 °C. The amplitude and rate constant were RNA = 48%, k = 0.28 min^–1^; DNA = 95%, k = 0.25 min^–1^. *D*, PAGE-based unwinding assay under the same conditions as in *panel C*. Reaction products were resolved by 15% native PAGE. Quantified band intensities (ImageJ) yielded the following parameters: RNA (•) = 47%, k = 0.25 min^–1^; DNA (○) = 84%, k = 0.24 min^–1^. In both (*C*) and (*D*), amplitude is normalized to heat-denatured controls (95 °C). Data represent means ± SD from five independent experiments. Substrate designs are provided in Table S1.

nsP13 unwinds DNA substrates more efficiently than dsRNA substrates (20, 23). Although the difference in unwinding activity between dsRNA and dsDNA substrates was attributed to the differences in binding affinity, limited studies have been conducted on the mechanism by which ATP hydrolysis affects binding affinity. In this study, dsDNA was used as the substrate to observe the single-turnover kinetics of dsDNA unwinding and ATP hydrolysis. The results were compared with the binding affinity of nsP13 to ssDNA substrates. In investigating the mechanism underlying dsDNA unwinding, nsP13 was purified with high purity (Fig. 1*A*). A schematic of the assay for dsDNA unwinding by nsP13 is shown in Figure 1*B*. In the presence of ATP, nsP13 bound to the 5′-ss tail without requiring ATP hydrolysis. Subsequently, ATP hydrolysis was initiated by adding magnesium ions and an excessive amount of TrapDNAs, which are non-fluorescent oligonucleotides with the same sequence as the duplex bottom strand (Table S1), and nsP13 started to translocate in the 5′–3′ direction with dsDNA unwinding. The displaced DNA strand was prevented from reannealing with the bottom strand because of TrapDNA (23, 26). Then, the unwound fluorescent DNA with different emission wavelengths was monitored based on the loss of fluorescence resonance energy transfer (FRET).

The concentration of the ATP was fixed while varying the magnesium ions. First, the optimal magnesium ion concentration at 3 mM ATP was determined (Fig. S1). At 3 mM ATP, most dsDNA was unwound at a magnesium ion concentration of 5 mM. Then, the unwinding of dsDNA and dsRNA substrates by nsP13 was observed (Fig. 1*C*). The time course of dsDNA or dsRNA unwinding was plotted and fitted to an exponential function to calculate the reaction amplitude and rate constant. The nsP13 helicase unwound 95% of the dsDNA substrates but only 48% of the dsRNA substrates. The nsP13 helicase unwound 95% of dsDNA substrates but only 48% of dsRNA substrates at 3 mM ATP. The separation of the double-stranded nucleic acids observed in a PAGE-based unwinding assay was compared with the results from a designed FRET-based unwinding assay. The nsP13 helicase efficiently unwound both dsDNA and dsRNA substrates, generating corresponding single-stranded products (ssDNA and ssRNA), which were subsequently resolved by 15% native PAGE (Figs. 1*D*, S1*C*). Upon unwinding, a downward shift in the fluorescein-labeled band was observed, consistent with successful strand separation. Notably, the absence of a reannealed duplex band indicates that the unlabeled trapDNA effectively hybridized with the displaced bottom strand, thereby preventing reannealing and stabilizing the single-stranded fluorescein-labeled product. For dsDNA and dsRNA, the amount of unwound duplex nucleic acids increased over time, with dsDNA being unwound more extensively. The PAGE-based unwinding assay results were consistent with those of the FRET-based unwinding assay.

To directly compare ATP requirements for duplex unwinding, we first performed reactions using 3 mM ATP under identical conditions for both dsDNA and dsRNA substrates. While dsDNA was almost completely unwound, dsRNA exhibited only partial unwinding. To determine whether this limited unwinding was due to ATP insufficiency, we increased the ATP concentration to 9 mM and repeated the unwinding and ATPase assays using the dsRNA substrate. As shown in Figure S2*A*, complete dsRNA unwinding was achieved at 9 mM ATP. Corresponding ATPase measurements (Fig. S2*B*) revealed that approximately 6 mM of the 9 mM ATP was hydrolyzed before the reaction plateaued, whereas nearly all 3 mM ATP was consumed during dsDNA unwinding. These results suggest that while dsRNA unwinding by nsP13 does not fully deplete the available ATP, it requires a higher total ATP input than dsDNA to achieve complete duplex separation, reflecting a greater energetic demand for processing RNA substrates.

### Directional unwinding of tailed duplex DNA and ssDNA-stimulated ATP hydrolysis

**Figure 2:**
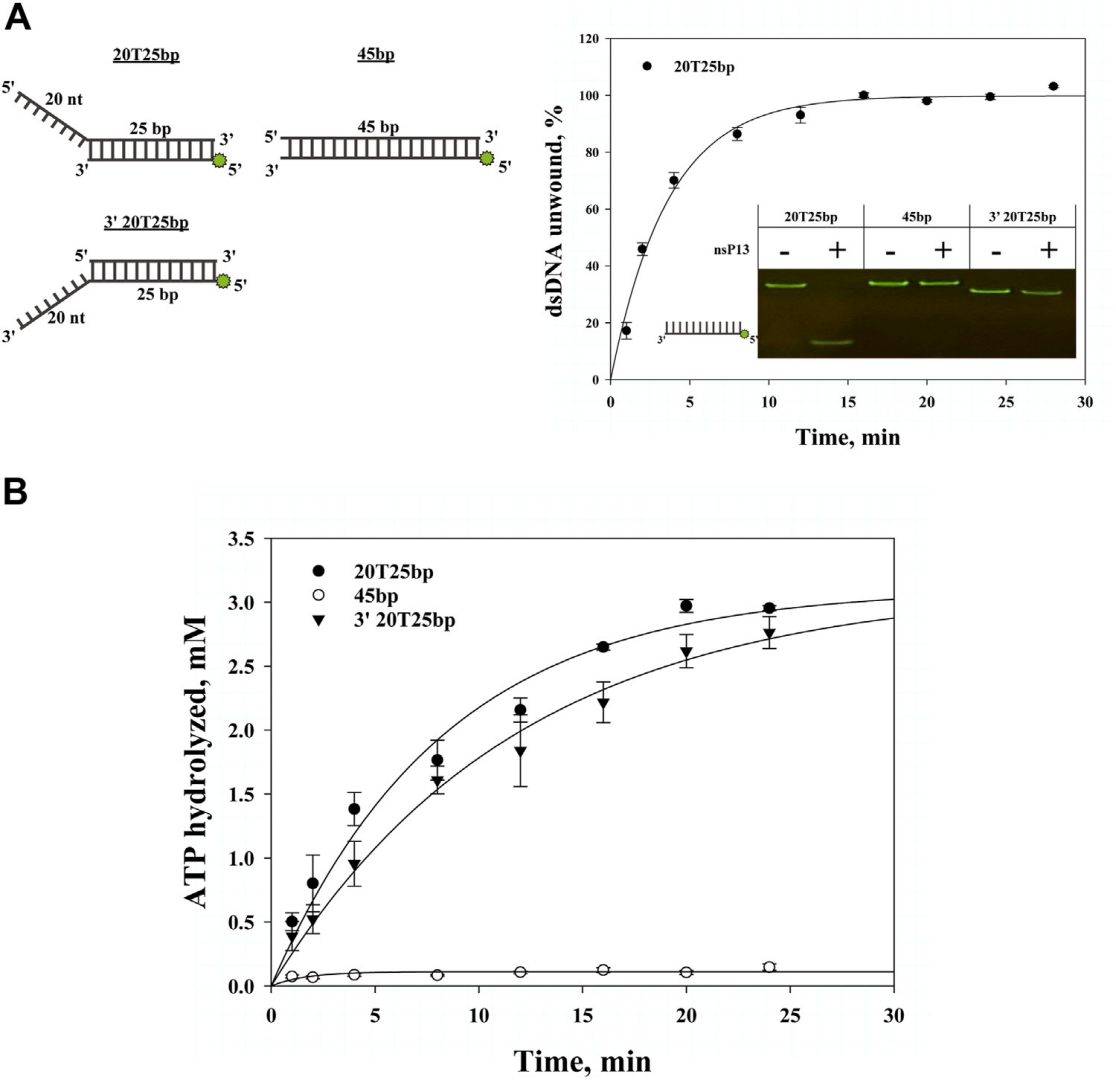
**Effects of 5′-tail polarity on unwinding and ATPase activity of nsP13.***A*, Time course of FRET-based unwinding of various dsDNA substrates at 37 °C. Gel insets show results after 20 min. Reactions were fitted to Equation 1. For 20T25 bp (•): amplitude = 100%, k = 0.27 min^–1^. *B*, colorimetric ATPase assay performed under the same conditions as in (*A*). ATP hydrolysis rate constants: 20T25 bp (•) = 0.17 min^–1^; 45 bp (○) = 0.07 min^–1^; 3′-20T25 bp (▼) = 0.14 min^–1^. Data are means ± SD.

In investigating dsDNA unwinding by nsP13 through ATP hydrolysis, unwinding assays and colorimetric ATP hydrolysis assays were performed using three substrates (20T25 bp, 45 bp, and 3′ 20T25 bp). In a previous study, experiments measuring the amount of unwound dsDNA over time using various types of dsDNA by nsP13 revealed that only dsDNA with a 5′-ss tail could be selectively unwound (26). In this study, duplex DNA substrates with blunt ends or a 3′-ss tail were not unwound, whereas those with a 5′-ss tail were unwound, as analyzed using 15% nondenaturing PAGE. In the FRET-based unwinding assay, ssDNA products were measured only for duplex DNA substrates with a 5′-ss tail (Fig. 2*A*).

To assess the relationship between ATP hydrolysis and helicase activity of nsP13, we performed ATPase assays using substrates containing either a 5′- or 3′-single-stranded DNA overhang (20T25 bp, 45 bp, and 3′20T25 bp; Fig. 2*B*). We observed that ATP hydrolysis occurred with both substrate types, indicating that the presence of a single-stranded DNA region is sufficient to stimulate the ATPase activity of nsP13. However, when these data were compared with duplex unwinding results obtained under the same conditions, a clear strand polarity-dependent effect was observed. While the 5′-tailed substrate supported both ATP hydrolysis and efficient duplex unwinding, the 3′-tailed substrate supported ATP hydrolysis but showed no detectable unwinding activity (Fig. 2, *A* and *B*). These findings suggest that while nsP13 can engage in ATP hydrolysis regardless of substrate orientation—possibly through cycles of binding, translocation, and dissociation—the presence of a 5′-ssDNA tail is specifically required to support productive unwinding. This functional asymmetry aligns with previous reports of 5′–3′ directional translocation by nsP13 (20, 26) and highlights the importance of substrate architecture in enabling helicase activity.

To examine whether the ATPase activity of nsP13 is modulated by single-stranded nucleic acids (ssNAs) and considering that nsP13 exhibits differential unwinding efficiency between RNA and DNA duplex substrates, we measured the initial rates of ATP hydrolysis in the presence of ssDNA and ssRNA across a range of ATP concentrations (Fig. S3). The apparent K_m_ values for ATP were similar for both ssDNA and ssRNA conditions (approximately 0.10 mM and 0.11 mM, respectively), indicating that, in the presence of ATP, nsP13 exhibits no significant difference in ATP-binding affinity toward either ssDNA or ssRNA.

### Docking of ATP and ATPγS into the nucleotide-binding site of nsP13

**Figure 3:**
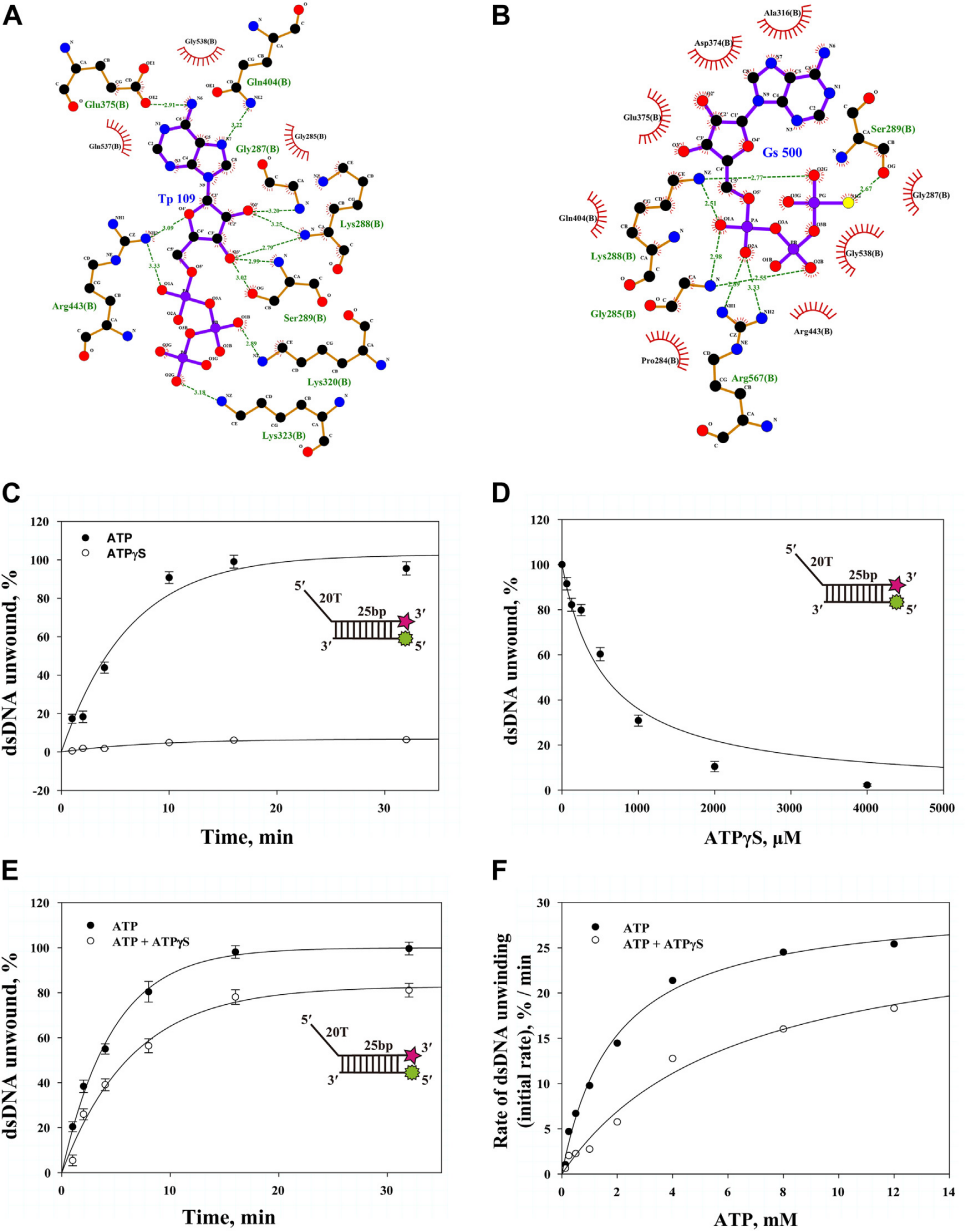
**Inhibition of nsP13 helicase activity by ATPγS.***A and B*, LigPlot + docking of nsP13 with ATP or ATPγS. Hydrogen bonds (*green dashed lines*) and hydrophobic interactions (*red lines*) are shown. *C*, time-dependent dsDNA unwinding with 3 mM ATP (•) or ATPγS (○). Amplitude and rate constant: ATP = 100%, k = 0.24 min^–1^; ATPγS = 7%, k = 0.14 min^–1^. *D*, dsDNA unwinding in the presence of 3 mM ATP and varying concentrations of ATPγS (0–60 nM). Increase in ATPγS diminished the amount of dsDNA unwound by nsP13 with a K_1/2_ of 0.58 mM ATPγS, as obtained by a hyperbolic fitting. *E*, inhibition of unwinding by 0.6 mM ATPγS at multiple ATP concentrations. At 4 mM ATP: ATP alone (•) = 100%, k = 0.26 min^–1^; ATP + ATPγS (○) = 78%, k = 0.16 min^–1^. Additional data are in Figure S4. *F*, initial unwinding rates as a function of ATP concentration ± 0.6 mM ATPγS. V_max_: ATP (•) = 30% unwound/min, K_1_/_2_ = 1.9 mM; ATP + ATPγS (○) = 28% unwound/min, K_1_/_2_ = 6.2 mM. All data represent means ± SD.

The translocation of nsP13, driven by the domain rotation induced by ATP binding and ATP hydrolysis, indicates the necessity for a continuous supply of ATP to support helicase translocation (27, 28). We hypothesized that disrupting ATP hydrolysis using a non-hydrolyzable analog such as ATPγS would impair the ATPase cycle of nsp13, thereby altering its ability to translocate along nucleic acids and unwind duplex DNA. This approach allowed us to specifically assess how ATPγS influences the conformational and mechanistic dynamics of nsP13 during helicase activity. In this study, a slowly hydrolyzable ATP analog, namely, ATPγS, was used to represent a state where ATP hydrolysis by nsP13 is inhibited. First, a docking study was conducted to observe the residues of nsP13, which interact with ATP and ATPγS. Docking analyses were performed to elucidate the binding sites and interacting amino acid residues of ATPγS in comparison with ATP (Fig. 3, *A* and *B*). The binding sites of ATP and ATPγS on nsP13 formed at the same location for ATP and ATPγS (Fig. S4). The nsP13 residues that interact with the beta and gamma phosphates of ATP include Lys320 and Lys323, which form hydrogen bonds around the phosphate chain (Fig. 3*A*). In addition, the nsP13 residues that interact with the beta and gamma phosphates of ATPγS include Gly285, Ser289, and Gln404, forming hydrogen bonds around the phosphates, as well as G287, K443, and G538, contributing to hydrophobic interactions (Fig. 3*B*). This finding indicates that ATPγS induces different interaction dynamics, although it binds at a site overlapping with ATP, thereby affecting the functionality of nsp13.

### Unwinding of duplex DNA with a fixed tail length in the presence of ATPγS

In the presence of ATP, dsDNA was completely unwound over time, while the maximum amount of dsDNA unwound was limited to approximately 7% in the presence of ATPγS. These results indicate that ATPγS is likely to inhibit the translocation activity of nsP13 during duplex DNA unwinding (Fig. 3*C*). Following this observation, an additional unwinding assay was conducted using varying concentrations of ATPγS to determine whether increasing levels of ATPγS could suppress dsDNA unwinding even in the presence of ATP (Fig. 3*D*).

With the increase of ATPγS concentration, the amount of unwound dsDNA decreased, with a 40% reduction at a concentration of 0.5 mM ATPγS. After confirming that ATPγS can act as an inhibitor, a dsDNA unwinding inhibition assay was conducted to determine whether ATPγS serves as a competitive inhibitor of ATP (Fig. 3*E*). The assay was performed at various concentrations of ATP, with Figure 3*E* showing 4 mM ATP, and the additional concentrations are presented in Figure S5. Comparing the amount of unwound dsDNA at 4 mM ATP with or without ATPγS, all dsDNA was unwound in the absence of ATPγS, whereas only 78% of the dsDNA was unwound in the presence of ATPγS (Fig. 3*E*).

The dsDNA unwinding at various ATP concentrations was fitted to an exponential curve, and the product of the amplitude and k (rate constant) was defined as the initial reaction rate (unwinding rate of reaction) at the corresponding ATP concentration. The initial reaction rate for each ATP concentration was fitted to a hyperbolic curve (Fig. 3*F*). The hyperbolic curve fitting values in accordance with the ATP concentrations reveal that an excessive amount of ATP in the presence of ATPγS does not affect the initial rates of dsDNA unwinding (V_max_ was similar regardless of ATPγS). However, K_1/2_, which represents the affinity of nsP13 for ATP, was 1.9 mM in the absence of ATPγS, but it increased by about 3.2-fold to 6.2 mM in the presence of ATPγS. This result indicates that ATPγS serves as a competitive inhibitor of ATP. Using the Cheng–Prusoff equation, which estimates the affinity of a competitive inhibitor, the K_i_ value was calculated to be 0.26 mM. This value is lower than the K_1_/_2_ of ATP, indicating that ATPγS binds more tightly to nsP13 under the experimental conditions.

### Unwinding of duplex DNA with varying tail lengths in the presence of ATPγS

**Figure 4:**
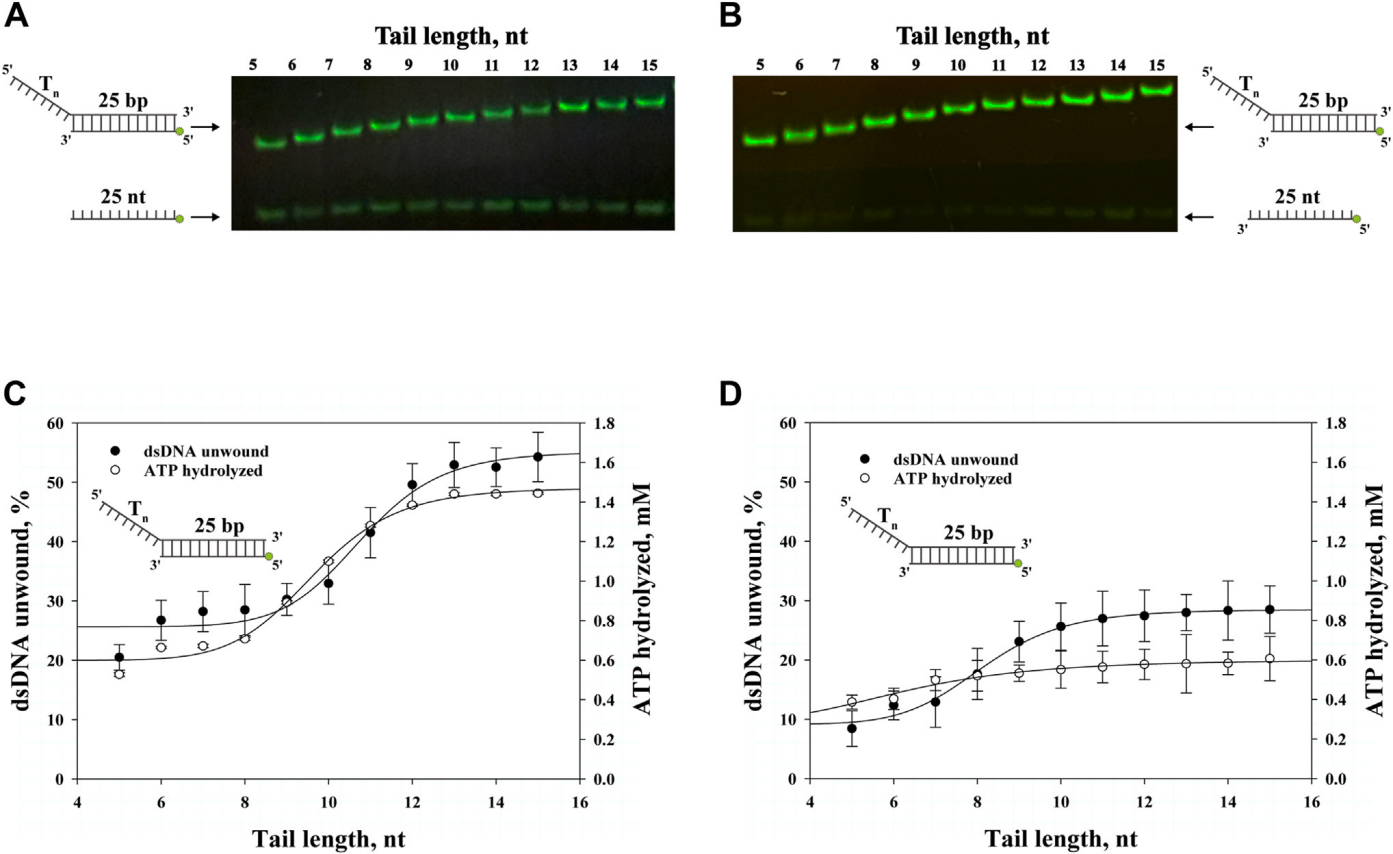
**Effect of 5′-tail length on dsDNA unwinding and ATP hydrolysis by nsP13.***A*, unwinding of Tn25 bp dsDNA substrates (various tail lengths) at 37 °C for 5 min. *B*, same as (*A*), in the presence of 0.6 mM ATPγS. *C*, correlation between dsDNA unwinding and ATP hydrolysis (same conditions as *A*). Data fit to Equation 3. For unwinding (•): A = 30%, y_0_ = 25%, C = 10 nts; ATP hydrolysis (•): A = 0.87 mM, y_0_ = 0.60 mM, C = 10 nts. *D*, same analysis as (*C*) under ATPγS conditions. For unwinding (•): A = 19%, y_0_ = 9.1%, C = 8.1 nts; ATP hydrolysis (○): A = 0.31 mM, y_0_ = 0.28 mM, C = 6.3 nts. All data are presented as means ± SD.

In this study, whether the amount of dsDNA unwound by nsP13 and the amount of hydrolyzed ATP increased with the length of the 5′-ss tail was investigated. To confirm this correlation, dsDNA substrates were designed with 5′-ss tails ranging from 5 to 15 nt in length (Table S1). A PAGE-based unwinding assay was conducted under two conditions (ATP and ATP + ATPγS) to examine the unwinding of dsDNA with varying 5′-ss tail lengths (Fig. 4, *A* and *B*). The amount of unwound dsDNA increased as the length of the 5′-ss tail increased (Fig. 4*A*). However, when ATPγS was added with ATP, the amount of unwound dsDNA increased with the increase of 5′-ss tails, but such an increase was lower compared with ATP alone (Fig. 4*B*).

After confirming dsDNA unwinding based on the length of the 5′-ss tail, the amount of ATP hydrolyzed by nsP13 on dsDNA substrates with varying 5′-ss tail lengths was measured (Fig. 4, *C* and *D*). In the absence of ATPγS, the amount of hydrolyzed ATP increased with the increase of the 5′-ss tail (Fig. 4*C*). However, in the presence of ATPγS, the amount of hydrolyzed ATP remarkably decreased compared with that in the absence of ATPγS as the length of the 5′-ss tail increased (Fig. 4*D*). Despite the presence of longer 5′-ss tails, the observed decrease in the amount of unwound dsDNA and hydrolyzed ATP in the presence of ATPγS is consistent with a previous study, in which the amount of unwound dsRNA decreased even with extended 5′-ss tails (23). As demonstrated in our previous study (23), high ATP concentrations promote cooperative translocation of multiple nsP13 monomers during duplex RNA unwinding. In our current study, we observed that in the presence of ATPγS, both dsDNA unwinding and ATP hydrolysis are significantly reduced, even when longer 5′-ss tails are present—conditions that favor the binding of multiple helicase monomers. This suggests that ATPγS impairs cooperative translocation, consistent with our previous findings and that this inhibitory effect persists even under conditions supporting multimeric helicase activity.

### The affinity of ssDNA binding by nsp13 is increased by ATP binding but is reduced upon ATP hydrolysis

**Figure 5:**
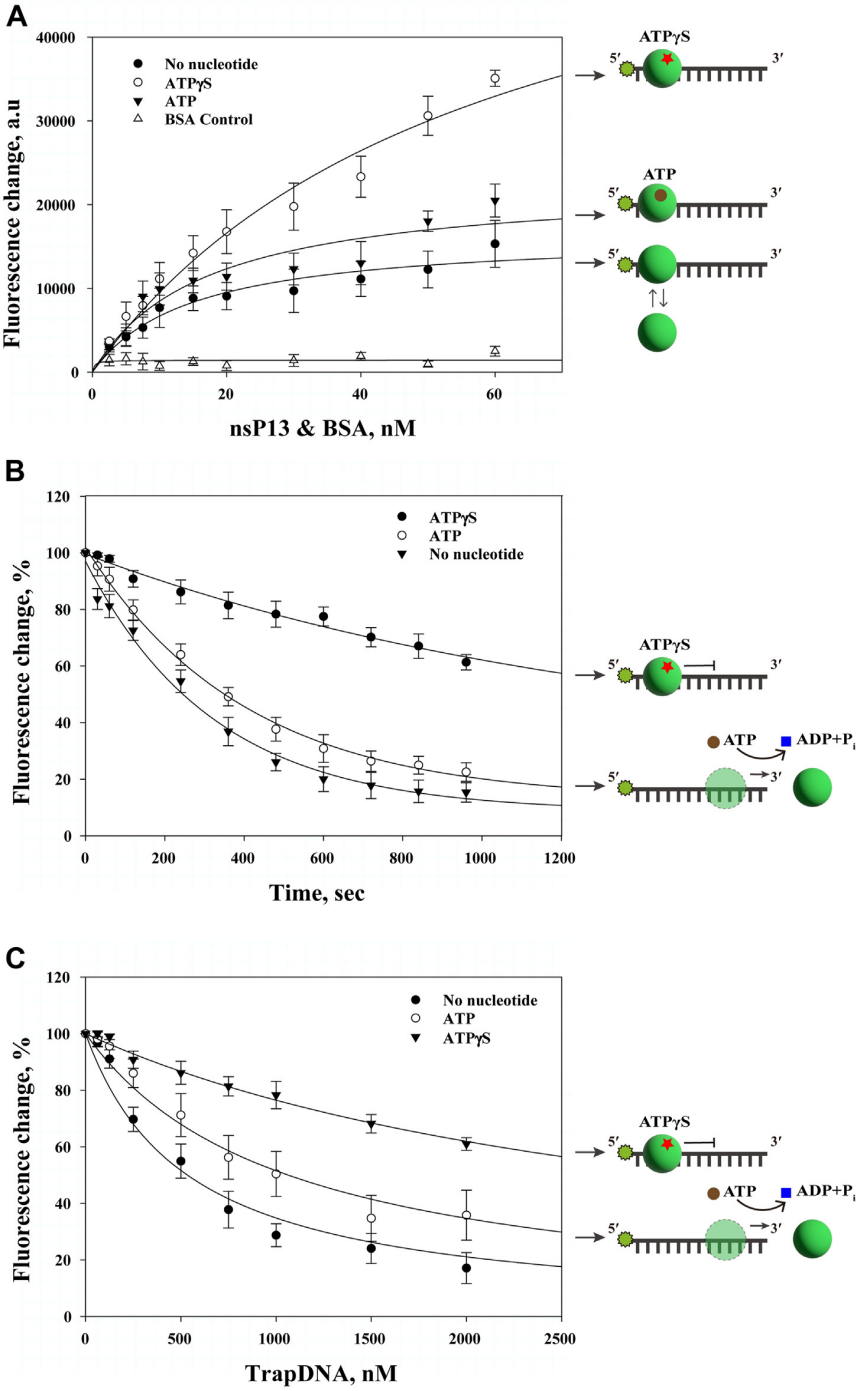
**ATP binding, not hydrolysis, promotes ssDNA binding by nsP13.***A*, fluorescence anisotropy of nsP13 binding to 25-nt fluorescein-labeled ssDNA-F (500 nM) in the presence of ATPγS (○), ATP (▼), or no nucleotide (•). BSA served as a negative control (Δ). Fluorescence increase with increasing protein concentration was fit to the hyperbolic equation (Eq. 2). Amplitudes and K_1/2_ in arbitrary fluorescence units: ATPγS amplitude = 65,000 a.u, K_1/2_ = 47.7 nM; ATP amplitude = 23,000 a.u, K_1/2_ = 17.7 nM; no nucleotide amplitude = 19,000 a.u, K_1/2_ = 15.6 nM; BSA = 1400 a.u, K_1/2_ value is not reliable. *B*, time-dependent displacement of ssDNA-F by TrapDNA (500 nM) in the presence of nsP13 (30 nM). Rate constants were obtained by fitting to the exponential decay: ATPγS (•) = 0.001 s^–1^; ATP (○) = 0.002 s^−1^; no nucleotide (▼) = 0.003 s^–1^. *C*, binding affinity measured by TrapDNA titration. Data were fit to a hyperbolic decay, and the fit provides K_1/2_ of TrapDNA for the fluorescence reduction: ATPγS (•) = 3100 nM; ATP (○) = 950 nM; no nucleotide (▼) = 460 nM. Data represent means ± SD.

When nsP13 binds to ATP, the domain rotation of the helicase induces the formation of a closed form, resulting in stronger binding to single-stranded nucleic acids (27, 29). We hypothesized that when ATP binds to nsP13, hydrolysis does not increase its binding affinity to ssDNA. However, when ATPγS binds to nsP13 without being hydrolyzed, the binding affinity of nsP13 to ssDNA increases. In this study, an nsP13-ssDNA binding assay was conducted on 25-nt ssDNA-F under three conditions (no nucleotide, ATP, and ATPγS; Fig. 5*A*). To determine whether the helicase binds to ssDNA in the absence of nucleotides, bovine serum albumin (BSA) was used as a negative control, and fluorescence was measured in the absence of nucleotides. Although no changes in fluorescence signals were observed with the increase of BSA concentration, the fluorescence signals increased with the increase of nsP13 concentration.

The fluorescence enhancement observed upon nsP13 binding to 5′-end-labeled ssDNA (ssDNA-F) likely results from changes in the local environment of the fluorophore. Such changes—often due to conformational alterations or reduced solvent exposure—are known to increase fluorescence intensity. We hypothesized that nsP13 binding induces structural rearrangements or shielding of the fluorescein moiety, thereby enhancing its emission. To validate this interpretation, we performed electrophoretic mobility shift assays (EMSA), which confirmed direct interaction between nsP13 and ssDNA-F, as evidenced by a clear mobility shift. Notably, the shift was more pronounced in the presence of ATPγS, indicating enhanced complex stability (Fig. S6*A*). Complementary absorbance measurements (Fig. S6*B*) showed increased absorbance near 495 nm upon nsP13 binding, with signal intensities rising by approximately 25% in the presence of ATP and nearly 50% with ATPγS, relative to the DNA-only control. Thus, the observed amplification of the fluorescence signal may indicate that nsP13 binding modifies the fluorophore’s environment, promoting greater light absorption and thus stronger fluorescence. This finding further demonstrates that nsP13 can bind to ssDNA even in the absence of nucleotides. ATP was used to examine the binding of nsP13 to ssDNA in the presence of nucleotides. Similar to the absence of a nucleotide, when nsP13 bound to ATP, the amount of nsP13 binding to ssDNA increased as the concentration of nsP13 increased. Furthermore, ATPγS was used to assess the binding of nsP13 to ssDNA in the absence of ATP hydrolysis. When nsP13 was bound to ATPγS, an increase in nsP13 concentration corresponded to a greater amount of nsP13 binding to ssDNA. Notably, nsP13 exhibited the highest level of binding under this condition compared with the other conditions.

To examine the nucleotide dependence of nsP13 binding to single-stranded DNA (ssDNA), we first conducted a fluorescence-based titration assay using a high concentration (5.0 μM) of 5′-fluorescein-labeled 25-mer ssDNA (ssDNA-F), which is 10-fold higher than the concentration used in previous experiments (Figs. 5*A*; S7). Under these conditions, the fluorescence signal increased with a relatively shallow saturation profile, likely reflecting the excess ssDNA relative to protein. To more accurately assess binding stoichiometry and achieve clearer saturation behavior, we repeated the titration assay using a lower ssDNA-F concentration (50 nM) while titrating increasing amounts of nsP13 (Fig. S8). This lower substrate concentration enabled more distinct observation of the binding curve and facilitated estimation of the minimal nsP13 concentration required to saturate the available binding sites. Fluorescence signals increased in a saturable manner across all conditions tested—ATPγS, ATP, and no nucleotide—indicating the formation of nsP13–ssDNA complexes. The binding curves plateaued between 100 and 125 nM nsP13, suggesting that approximately 2 to 2.5 molecules of nsP13 can occupy each ssDNA molecule. This estimate aligns with previous reports that one nsP13 monomer spans ∼10 to 12 nucleotides (26). Importantly, the amplitude of fluorescence and the apparent K_1/2_ values varied depending on the nucleotide condition. Binding in the presence of ATPγS showed both a higher signal amplitude and a lower K_1/2_ compared to ATP or the nucleotide-free condition, indicating enhanced binding affinity and complex stability. While ATP and no nucleotide conditions produced similar K_1/2_ values, the extent of fluorescence enhancement was significantly greater with ATPγS. These results suggest that ATPγS promotes a closed, high-affinity conformation of nsP13, facilitating complex formation at lower protein concentrations.

Next, we investigated whether nsP13 binds to ssDNA in the presence of ATPγS and remains bound for a longer duration. In confirming the dissociation of nsP13 from ssDNA over time, a competitive kinetic dissociation assay in the presence of TrapDNA was performed (Fig. 5*B*). In the absence of nucleotides, the fluorescence signal decreased over time, indicating that nsP13 dissociated from ssDNA over time. When ATP was bound to nsP13, the dissociation pattern was similar to that observed in the absence of nucleotides. However, when ATPγS was bound to nsP13, it was observed that nsP13 dissociated more slowly than when under other conditions (Fig. 5*B*). Therefore, ATPγS allows nsP13 to remain bound to ssDNA for a longer duration.

Overall, this study aimed to determine the cause of the slow dissociation of nsP13 from ssDNA in the presence of ATPγS by observing the binding affinity between nsP13 and ssDNA. In measuring binding affinity, the dissociation of nsP13 was observed based on the concentration of TrapDNA (Fig. 5*C*). In the absence of nucleotides, the fluorescence signal decreased remarkably with the increase of TrapDNA concentration. In the absence of nucleotides, the fluorescence signal decreased the most as the TrapDNA concentration increased compared with other conditions. This phenomenon indicates that nsP13 can easily dissociate from ssDNA without nucleotides. In the presence of ATP, nsP13 also dissociated from ssDNA as the TrapDNA concentration increased, but the decrease was less pronounced than that when nucleotides are absent. However, in the presence of ATPγS, nsP13 exhibited the least dissociation from ssDNA as the TrapDNA concentration increased. Therefore, when nsP13 binds to nucleotides, its binding affinity to ssDNA increases.

## Discussion

SARS-CoV-2 nsP13 unwinds DNA and RNA substrates in the 5′–3′ direction using the energy released by ATP hydrolysis (12, 21, 23, 28). These results indicate that ATP hydrolysis by nsP13 is essential for dsDNA unwinding (13). Although dsDNA is not the native substrate of nsP13 *in vivo*, its use in this study is justified by the helicase’s demonstrated ability to unwind both RNA and DNA duplexes with a 5′ to 3′ polarity, and the observation that both ssRNA and ssDNA similarly stimulate its ATPase activity. These findings underscore the enzyme’s substrate flexibility and support the relevance of DNA-based assays for mechanistic exploration. Duplex DNA provides a structurally defined and experimentally robust system that enables precise dissection of the helicase’s unwinding mechanism, including the identification of factors that modulate its activity. Importantly, the mechanistic insights gained from studies using dsDNA can inform our understanding of how nsP13 functions under diverse physiological and stress-related conditions, where alternative nucleic acid structures may be encountered. Thus, while RNA is the physiological substrate, studying DNA unwinding serves as a valuable model to uncover general principles of nsP13 helicase activity.

A previous study has demonstrated that helicases are less efficient at unwinding dsRNA substrates compared with dsDNA substrates (23). However, the amount of unwound dsRNA increased with the increase in ATP concentration. In this study, the amount of unwound dsDNA substrates increased with the amount of hydrolyzed ATP. However, when ATPγS was used, the amount of unwound dsDNA decreased, even in the presence of ATP. Similarly, the amount of ATP hydrolyzed by nsP13 also decreased under these conditions. These findings indicate that inhibiting the ATP hydrolysis activity of nsP13 can effectively suppress duplex nucleic acid unwinding, thereby providing valuable insights into the discovery of compounds that inhibit helicase activity. Based on previous studies, nsP13 exhibited varying affinities to ssDNA depending on the nucleotide, indicating that ATP hydrolysis is a critical factor in determining the processivity of nsP13 during duplex unwinding (23). While it is well established that helicase activity requires ATP hydrolysis, our findings reveal that efficient duplex unwinding by nsp13 is not solely dependent on ATP presence but is closely linked to the hydrolysis rate. The markedly reduced unwinding activity observed with ATPγS—a slowly hydrolyzable analog—suggests that translocation by nsp13 is kinetically constrained by hydrolysis efficiency, emphasizing the importance of rapid turnover for effective strand separation.

Fluorescence-based analysis of nsP13 binding onto the fluorescently labeled ssDNA was performed using a 25-nt ssDNA substrate under three conditions (no nucleotide, ATP, and ATPγS). As shown in Figure 5*A*, fluorescence anisotropy caused by nsP13 binding to the fluorescently labeled ssDNA was measured to assess the binding affinity of nsP13 to a 25-nt ssDNA substrate in the presence of ATPγS. As the concentration of nsP13 increased, the fluorescence anisotropy signal increased in a concentration-dependent manner, indicating enhanced binding. ATP binding and hydrolysis induce structural changes in the domains of nsP13 (27). These findings are consistent with the previously observed results of RNA-nsP13 binding affinity to RNA substrates (23, 25). However, additional experiments are necessary to investigate the nucleotide-dependent binding affinity to RNA and DNA substrates.

The majority of non-ring-shaped SF1 helicases, such as SARS-CoV-2 nsP13, contain two nucleic acid binding sites, RecA-like domains, in their motor core domains (27). The helicase exists in a closed state induced by ATP binding as well as in an open state resulting from ATP hydrolysis and subsequent product release (27, 30). These conformational cycles are essential for the translocation of nsP13. Thus, structural changes occur in the helicase during ATP binding and hydrolysis. Upon ATP hydrolysis and product release, the helicase adopts an open state, enabling translocation. This phenomenon indicates that the increased binding affinity of nsP13 to ssDNA in the presence of ATPγS results from being in a closed state for a long period of time. However, considering that only ATP and ATPγS were used, studies on the binding affinity of nsP13 for ADP, which is a product of ATP hydrolysis, are lacking. Therefore, conducting additional research involving ADP is necessary to define and clarify the changes in binding affinity following ATP hydrolysis.

Previous studies have reported that HCV NS3 exhibited a lower binding affinity to nucleic acid substrates in the closed state than in the open state (31). Therefore, the binding affinity of the SARS-CoV-2 helicase for ATP and RNA is critical for its function, and maintaining specific interactions in the active complex is key to its catalytic activity, offering potential targets for inhibitor development (32). In this study, nsP13 exhibited higher binding affinity to DNA substrates and lower duplex unwinding activity with ATPγS than with ATP. This finding indicates that maintaining a closed state increases the binding affinity of nsP13, while cycling between the open and closed states during ATP hydrolysis may reduce binding affinity. Further experiments using ADP and P_i_ to assess duplex unwinding and binding affinity during different stages of ATP hydrolysis are necessary. The binding affinity of nsP13, which varies with ATP hydrolysis, can influence the duplex unwinding and translocation of nsP13.

Several studies have been conducted targeting nsP13 inhibitors. These studies have focused on identifying compounds that inhibit helicase duplex unwinding or ATP hydrolysis activity (30, 33, 34, 35). However, further research on helicase inhibitors is hindered by the low biocompatibility of these compounds for human use. Similarly, ATPγS, which we used in our study, is also unsuitable for human application. Therefore, for effective inhibitor development, designing compounds that inhibit ATP hydrolysis while mimicking the functional role of ATP is necessary.

Although extended 5′ single-stranded tails generally promote duplex DNA unwinding by providing additional binding sites for helicases (36), this enhancement is markedly suppressed in the presence of ATPγS. This inhibition likely arises because ATPγS-bound nsP13 remains in a non-hydrolyzing, tightly bound state, which prevents translocation along the ssDNA and limits progression into the duplex region. As a result, the generation of additional ssDNA is impaired, reducing opportunities for cooperative binding by multiple nsP13 molecules (36, 37). These findings suggest that while longer 5′-ss tails facilitate unwinding under normal hydrolytic conditions, ATPγS disrupts this effect by stabilizing nsP13 in a static, high-affinity binding conformation that hinders translocation and duplex separation.

ATP hydrolysis normally induces structural changes and cycling between the open and closed conformations, thereby facilitating translocation. By contrast, ATPγS failed to induce these structural changes, causing nsP13 to remain bound to ssDNA without translocation. ATP hydrolysis induces structural changes, enabling cycling between the open and closed states, thereby facilitating translocation. In addition, the binding affinity to single-stranded nucleic acids can increase in the closed conformation. This correlation indicates that maintaining the ATP binding and ATP hydrolysis cycle is crucial for nsP13 translocation. Therefore, inhibiting ATP hydrolysis by the helicase can disrupt the maintenance of the ATP binding and hydrolysis cycle. In this study, ATPγS causes nsP13 to remain bound to ssDNA for a longer duration and with stronger affinity. These results indicate that ATPγS prevents nsP13 from undergoing ATP hydrolysis, thereby disrupting the ATP binding and hydrolysis cycle, which inhibits the translocation of nsP13 and allows it to retain high binding affinity in a closed state.

**Figure 6:**
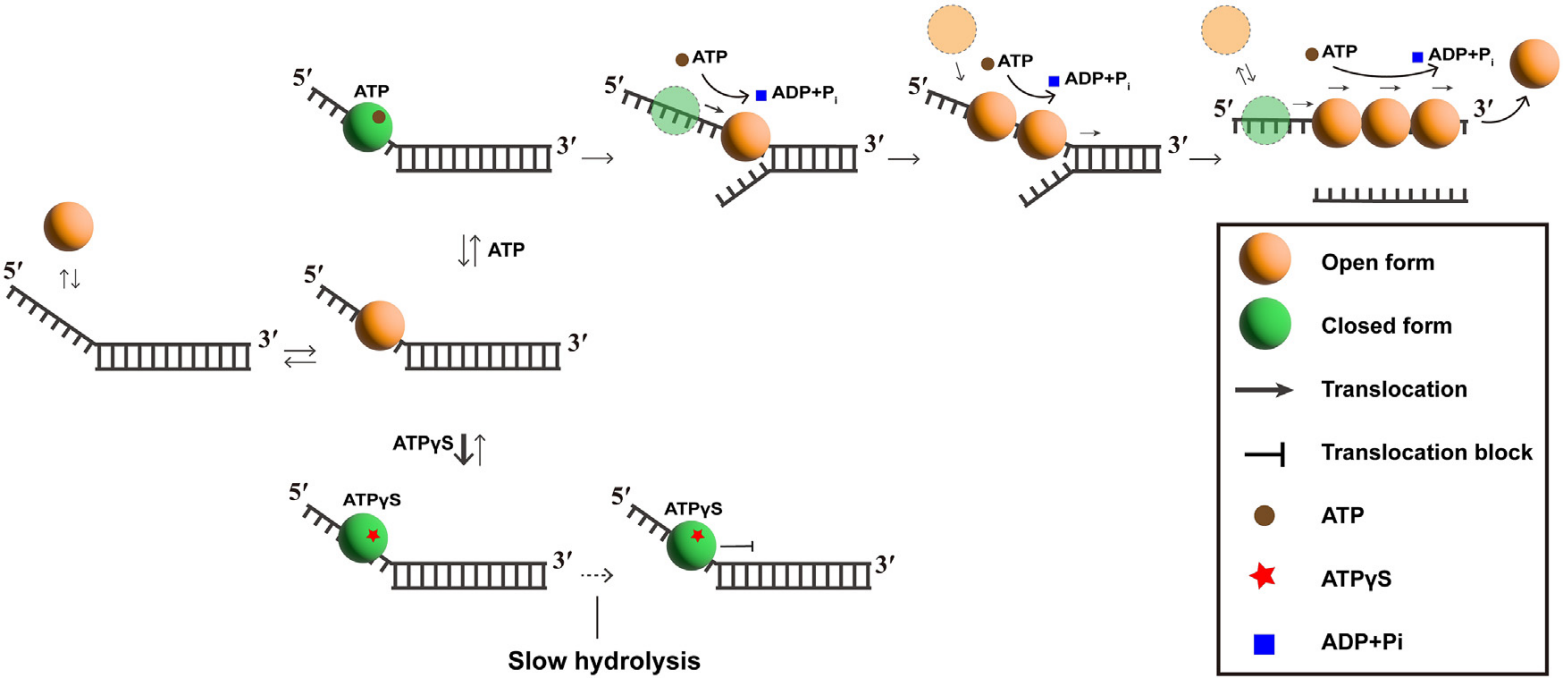
**Proposed mechanism of ATP hydrolysis–driven unwinding by the nsP13 helicase.** Schematic representation of the model whereby ATP binding promotes ssNA engagement and ATP hydrolysis facilitates translocation and duplex unwinding.

In this study, changes in the duplex nucleic acid unwinding activity of nsP13 and its binding affinity to ssDNA induced by ATP hydrolysis were observed. Furthermore, ATP binding to nsP13 enhanced its binding affinity to ssDNA. Moreover, the binding affinity between nsP13 and ssDNA was significantly higher in the presence of ATPγS than when ATP is present. This result indicates that ATP binding affects binding affinity during ATP hydrolysis. For translocation to occur, the binding affinity must decrease because of ATP hydrolysis. These findings indicate that the reduction in the binding affinity of nsP13 to ssDNA through ATP hydrolysis facilitates its translocation (Fig. 6). Therefore, ATP hydrolysis–dependent duplex DNA unwinding by SARS-CoV-2 nsP13 relies on efficient binding and translocation along single-stranded nucleic acids.

## Experimental procedures

### Preparation of the nsP13 protein and duplex nucleic acids

The gene encoding the SARS-CoV-2 helicase nsP13 domain was chemically synthesized and cloned into the expression plasmid DNA (LumiMac, Inc). The nsP13 protein was purified in accordance with previously established protocols (38). The ssDNA substrate was purchased from Bionics, and the ssRNA substrate was purchased from IDT. A 10-μl solution containing 20 mM TE buffer, 50 mM NaCl, and 5 μM single-stranded nucleic acid was mixed thoroughly to prepare duplex nucleic acids. The mixture was heated at 90 °C for 5 min and then incubated at 25 °C for 40 min to allow annealing.

### FRET-based assay of duplex nucleic acid unwinding

For standard RNA and DNA unwinding assays, mixture A (containing 15 nM nsP13 helicase, 20 mM HEPES [pH 7.4], 0.1 M NaCl, various concentrations of ATP, and 500 nM duplex nucleic acid) and mixture B (5 mM MgCl_2_ and 2.5 μM trap DNA or RNA, an unlabeled oligonucleotide identical in sequence to the bottom strand of duplex nucleic acid substrate) were preincubated for 5 min at 37 °C (23, 26). The unwinding reaction was initiated by mixing the two mixtures. All reactions were conducted at 37 °C at various times. The reactions were stopped by adding an equal volume of termination buffer (20 mM HEPES [pH 7.4], 0.2 M EDTA, and 0.2 M NaCl). A control for maximum unwinding was prepared by heating the duplex substrate at 95 °C for 15 min. Duplex nucleic acid unwinding was measured using a Victor III multiplate reader. The proportion of unwound RNA or DNA was fitted using the following single exponential equation:

(1) F(t)=A∗{1-exp(k∗t)}

where F(t) is the fraction unwound at time t; A is the amplitude of duplex nucleic acid unwinding, and k is the rate constant of duplex nucleic acid unwinding.

### PAGE-based assay of duplex nucleic acid unwinding

For standard RNA and DNA unwinding assays, mixture A (15 nM nsP13 helicase, 20 mM HEPES [pH 7.4], 0.1 M NaCl, 3 mM ATP, and 500 nM duplex nucleic acid) and mixture B (5 mM MgCl_2_ and 2.5 μM Trap oligo) were preincubated for 5 min at 37 °C. The unwinding reaction was initiated by mixing the two mixtures. All reactions were conducted at 37 °C at various times. The reactions were stopped by adding an equal volume of termination buffer (20 mM HEPES [pH 7.4], 0.2 M EDTA, and 0.2 M NaCl). The reaction result was confirmed using 15% nondenaturing PAGE. A control for maximum unwinding was prepared by heating the duplex substrate at 95 °C for 15 min and loading it onto the gel immediately. The gel was exposed to a UV/Vis transilluminator, and the band intensities were quantified using ImageJ. Amplitude represents the relative amount of unwound duplex nucleic acid, normalized to the value obtained after a 15-min reaction at 95 °C, which was set as 100. The proportion of unwound RNA or DNA was fitted using a single exponential equation (Eq. 1). F(t) is the fraction unwound at time t; A is the amplitude of unwinding, and k is the rate constant of duplex nucleic acid unwinding.

### Colorimetric ATP hydrolysis assay

The experiment measuring the amount of hydrolyzed ATP was conducted in accordance with the protocol provided by the Malachite Green Phosphate Assay Kit (Sigma-Aldrich Co., St Louis, MO, USA). The ATPase reaction involved the preincubation of mixture A (15 nM nsP13 helicase, 20 mM HEPES [pH 7.4], 0.1 M NaCl, 3 mM ATP, and 500 nM dsDNA) and mixture B (5 mM MgCl_2_ and 2.5 μM TrapDNA) for 5 min at 37 °C. The unwinding reaction was initiated by mixing the two mixtures. All reactions were conducted at 37 °C at various times. The reactions were stopped by adding an equal volume of termination buffer (20 mM HEPES [pH 7.4], 0.2 M EDTA, and 0.2 M NaCl). Each sample (4 μl) was mixed with 76 μl D.W. and 20 μl of malachite green dye in a 96-well plate, incubated for 30 min at 25 °C, and measured at 650 nm using a Victor III multiplate reader. The absorbance was converted to P_i_ concentration using a standard curve, and the unwinding kinetics were fitted using a single exponential equation (Eq. 1). Amplitude refers to the amount of hydrolyzed ATP, and the maximum amplitude corresponds to 3 mM. F(t) indicates the fraction of unwound at time t; A is the amplitude of unwinding, and k is the observed rate constant of duplex nucleic acid unwinding.

### Nucleotide docking simulation

The crystal structure of nsP13 (PDB ID: 7NIO) was retrieved from the Protein Data Bank and prepared by removing the water molecules and ligands. Nucleotides were docked to the nsP13 helicase using AMDock, with the docking site set to the nucleotide-binding region. The structures of ATP (DB00171) and ATPγS (DB02930) were minimized to ensure proper geometry before docking. After docking, the binding interactions were analyzed using LigPlot^+^ to identify the specific amino acid residues involved in hydrogen bonds, hydrophobic contacts, and electrostatic interactions.

### dsDNA unwinding in the presence of the ATP analog ATPγS

The unwinding assay was conducted in a reaction containing mixture A (15 nM nsP13 helicase, 20 mM HEPES [pH 7.4], 0.1 M NaCl, various concentrations of ATP, various concentrations of ATPγS, and 500 nM dsDNA) and mixture B (5 mM MgCl_2_ and 2.5 μM TrapDNA) preincubated for 5 min at 37 °C. The unwinding reaction was initiated by mixing the two mixtures. All reactions were conducted at 37 °C at various times. The reactions were stopped by adding an equal volume of termination buffer (20 mM HEPES [pH 7.4], 0.2 M EDTA, and 0.2 M NaCl). A control for maximum unwinding was prepared by heating the duplex substrate at 95 °C for 15 min. Duplex nucleic acid unwinding was measured using a Victor III multiplate reader. The proportion of unwound DNA was fitted using a single exponential equation (Eq. 1). The amplitude and k values of the exponential curve at each ATP concentration were multiplied to calculate the initial reaction rate, which was then fitted to the following hyperbolic equation:

(2) F(x)=(A∗x)/(B+x)

Where F(x) represents the initial rate as a function of ATP concentration x; A is the amplitude of the reaction rate, and B(K_1/2_) is the ATP concentration at which F(x) reaches half of its amplitude.

The unwinding assay was conducted in a reaction containing mixture A (15 nM nsP13 helicase, 20 mM HEPES [pH 7.4], 0.1 M NaCl, 3 mM ATP, 3 mM ATPγS, and 500 nM Tn25 bp) and mixture B (5 mM MgCl_2_ and 2.5 μM TrapDNA), which were preincubated for 5 min at 37 °C. The unwinding reaction was initiated by mixing the two mixtures. All reactions were conducted at 37 °C for 5 min. The reactions were stopped by adding an equal volume of termination buffer (20 mM HEPES [pH 7.4], 0.2 M EDTA, and 0.2 M NaCl). The reaction results were confirmed using 15% nondenaturing PAGE. The gel was exposed to a UV/Vis transilluminator, and the band intensities were quantified using ImageJ. The proportion of unwound DNA was fitted using the following sigmoidal equation:

(3) $$F\left(x\right)=y_{0}+\left(A\;*\;x^{b}\right)\;/\;\left(C^{b}+x^{b}\right)$$

where F(x) represents the amount of dsDNA unwound; y_0_ represents the baseline level of dsDNA unwound; A is the maximum increase in unwound dsDNA; C is the x-value corresponding to half of the maximum increase, and b determines the steepness of the curve. The ATP hydrolysis assay was conducted under the same conditions in accordance with the measurement method of the dsDNA unwinding inhibition assay by ATPγS.

### Fluorometric assay of ssDNA binding by nsP13

The binding assay was conducted in a reaction buffer containing various concentrations of nsP13 helicase, 20 mM HEPES (pH 7.4), 0.1 M NaCl, 3 mM ATP or ATPγS, and 500 nM 25-nt ssDNA-F. In initiating the reaction, 5 mM MgCl_2_ was added, and the mixture was incubated for 3 min at 25 °C. Fluorescence measurements were taken using a VICTOR III multiplate reader set to detect FITC fluorescence. Fluorescence data were fitted using Equation 2), where A is the amplitude of the fluorescence, and B (K_1/2_) is the nsP13 concentration at which F(x) reaches half of its amplitude. A reaction buffer containing 30 nM of nsP13 helicase, 20 mM HEPES (pH 7.4), 0.1 M NaCl, 5 mM MgCl_2_, 3 mM ATP or ATPγS, and 500 nM of 25-nt ssDNA-F was incubated for 3 min at 25 °C. After incubation, various concentrations of TrapDNA were added, and the mixture was incubated at 25 °C for various times. Fluorescence measurements were taken using a VICTOR III multiplate reader set to detect FITC fluorescence. The 100% value represents fluorescence before adding TrapDNA. The fluorescence data were fitted using the hyperbolic decay equation.

## Data availability

Please contact the corresponding authors at “kimde@konkuk.ac.kr”; for any of the raw data not provided in this manuscript.

## Supporting information

This article contains supporting information.

## Conflict of interest

The authors declare that they have no conflicts of interest with the contents of this article.
