# Supplementary material for: ATPase-dependent duplex nucleic acid unwinding by SARS-CoV-2 nsP13 relies on facile binding and translocation along single-stranded nucleic acid
Source: J Biol Chem. 2025 Jun 12;301(7):110373. doi: 10.1016/j.jbc.2025.110373 (PMC12274814; doi:10.1016/j.jbc.2025.110373)
Supplement: Supporting information [file mmc1.docx]

ATPase-dependent duplex nucleic acid unwinding by SARS-CoV-2 nsP13 relies on facile binding and translocation along single-stranded nucleic acid

Jinwoo Park^1,#^, Yong-Joo Jeong^2,#^, Khushbu Chauhan^1^, Hye Ran Koh^3^, and Dong-Eun Kim^1, *^

^1^Department of Bioscience and Biotechnology, Konkuk University, Seoul 05029, Republic of Korea; ^2^Department of Chemistry, Chung-Ang University, Seoul 06974, Republic of Korea

*To whom correspondence should be addressed: Dong-Eun Kim, Ph.D. Department of Bioscience and Biotechnology, Konkuk University, Seoul 05029, Republic of Korea. Tel: +82-2-2049-6062; Fax: +82-2-3436-6062; E-mail: [kimde@konkuk.ac.kr](mailto:kimde@konkuk.ac.kr)

# J.P. and Y.-J.J. equally contributed to this study.

**Supplementary Fig. S1**


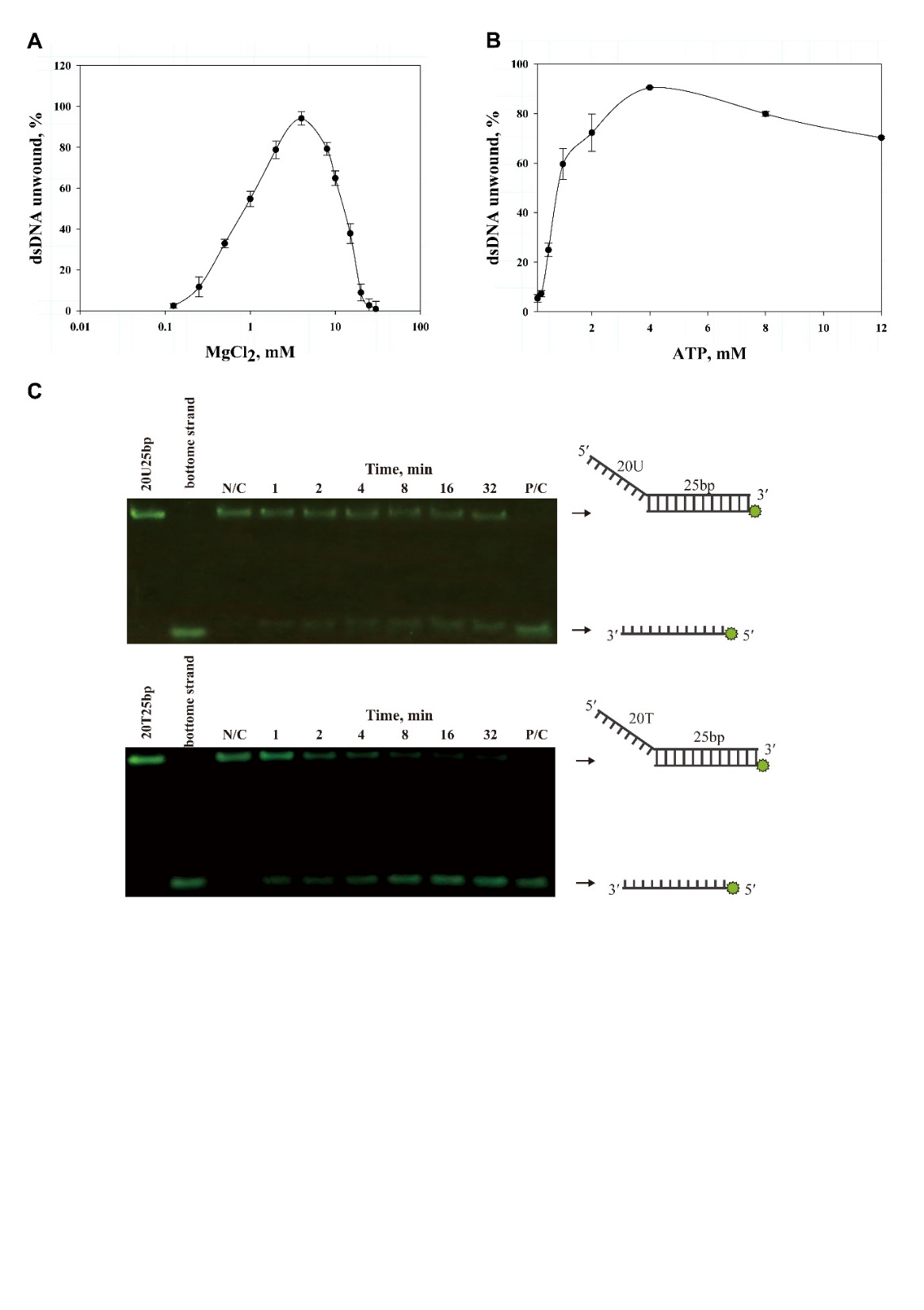


**Figure S1. dsDNA unwinding activity of nsP13 as a function of ATP and Mg²⁺ concentrations.** (A) Unwinding of 20T25bp-T dsDNA (500 nM) by nsP13 at varying MgCl₂ concentrations (0.125–30 mM) with a fixed ATP concentration (3 mM). Reactions were performed at 37°C for 10 min in the presence of trap DNA (2.5 μM; unlabeled oligonucleotide complementary to the bottom strand) to prevent reannealing of unwound products. (B) Unwinding of 20T25bp-T dsDNA (500 nM) at varying ATP concentrations in the presence of a fixed MgCl₂ concentration (5 mM) and trap DNA (2.5 μM). (C) Time-course analysis of duplex DNA and RNA unwinding by nsP13. Reactions were performed using 500 nM of either 20T25bp (DNA) or 20U25bp (RNA) substrates with 5 mM MgCl₂, 3 mM ATP, and 2.5 μM trap DNA at 37°C. Reaction products were resolved by 15% native PAGE. N/C, negative control (substrate only); P/C, positive control (heat-denatured duplex substrate in the presence of trap DNA). All experiments were independently repeated at least three times. Representative gel images are shown, and unwinding efficiencies were quantified using ImageJ. Error bars represent standard deviation where applicable.

**Supplementary Fig. S2**


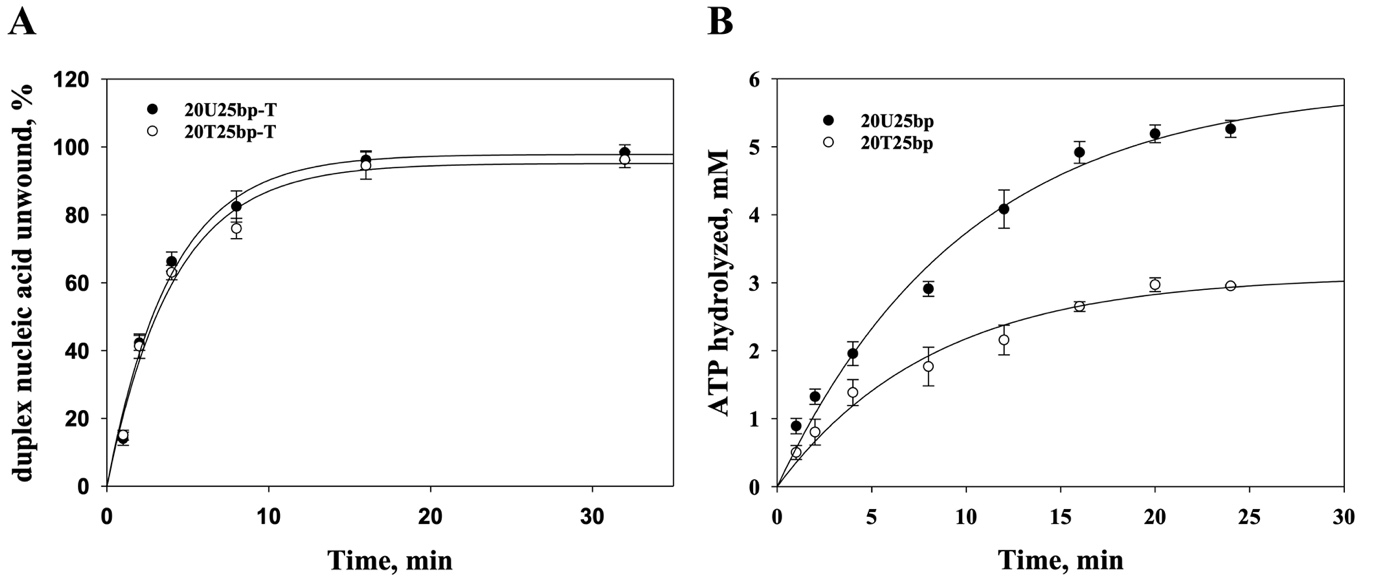


**Figure S2. Comparison of duplex unwinding and ATP hydrolysis between dsRNA and dsDNA substrates.** (A) Time-course analysis of duplex unwinding using 20U25bp-T (dsRNA) and 20T25bp-T (dsDNA) substrates at 37 °C. Reactions were carried out in the presence of 3 mM ATP for dsDNA and 9 mM ATP for dsRNA. Fluorescence signals were recorded using a VICTOR III plate reader and fitted to a single exponential rise-to-maximum equation. Fitted parameters were as follows: 20T25bp-T, amplitude = 99.1, rate constant (k) = 0.25; 20U25bp-T, amplitude = 99.9, k = 0.28. (B) ATP hydrolysis under the same conditions as in (A), measured using a colorimetric ATPase assay as described in the Materials and Methods. Absorbance values were recorded on a VICTOR III plate reader and fitted to the same exponential model. Fitted values: 20T25bp-T, amplitude = 2.92, k = 0.19; 20U25bp-T, amplitude = 5.47, k = 0.31.

**Supplementary Fig. S3**

*
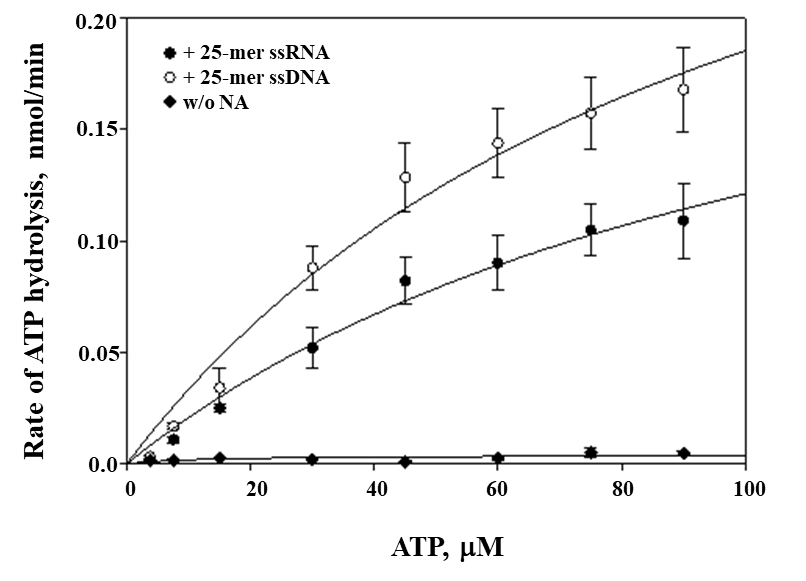
*

**Figure S3. Steady-state kinetics of SARS-CoV-2 nsP13 ATPase activity in the presence and absence of single-stranded nucleic acids.** ATP hydrolysis by nsP13 (15 nM) was assayed at 37 °C using increasing concentrations of ATP under three conditions: with 25-mer single-stranded DNA (ssDNA, ○), 25-mer single-stranded RNA (ssRNA, ●), and in the absence of nucleic acids (◆). Initial reaction velocities were derived from time-course measurements and fitted to the Michaelis–Menten equation to obtain kinetic parameters. In the presence of ssDNA, the apparent K_m_ for ATP was 0.103 ± 0.022 mM with a k_cat_ of 121 ± 26 min⁻¹. In the presence of ssRNA, the K_m_ was 0.107 ± 0.015 mM and the k_cat_ was 88.4 ± 16 min⁻¹. No measurable ATPase activity was observed in the absence of nucleic acids, confirming that single-stranded nucleic acids are required for nsP13 catalytic activation. Each data point represents the mean of at least three independent experiments; error bars indicate standard deviation.

**Supplementary Fig. S4**


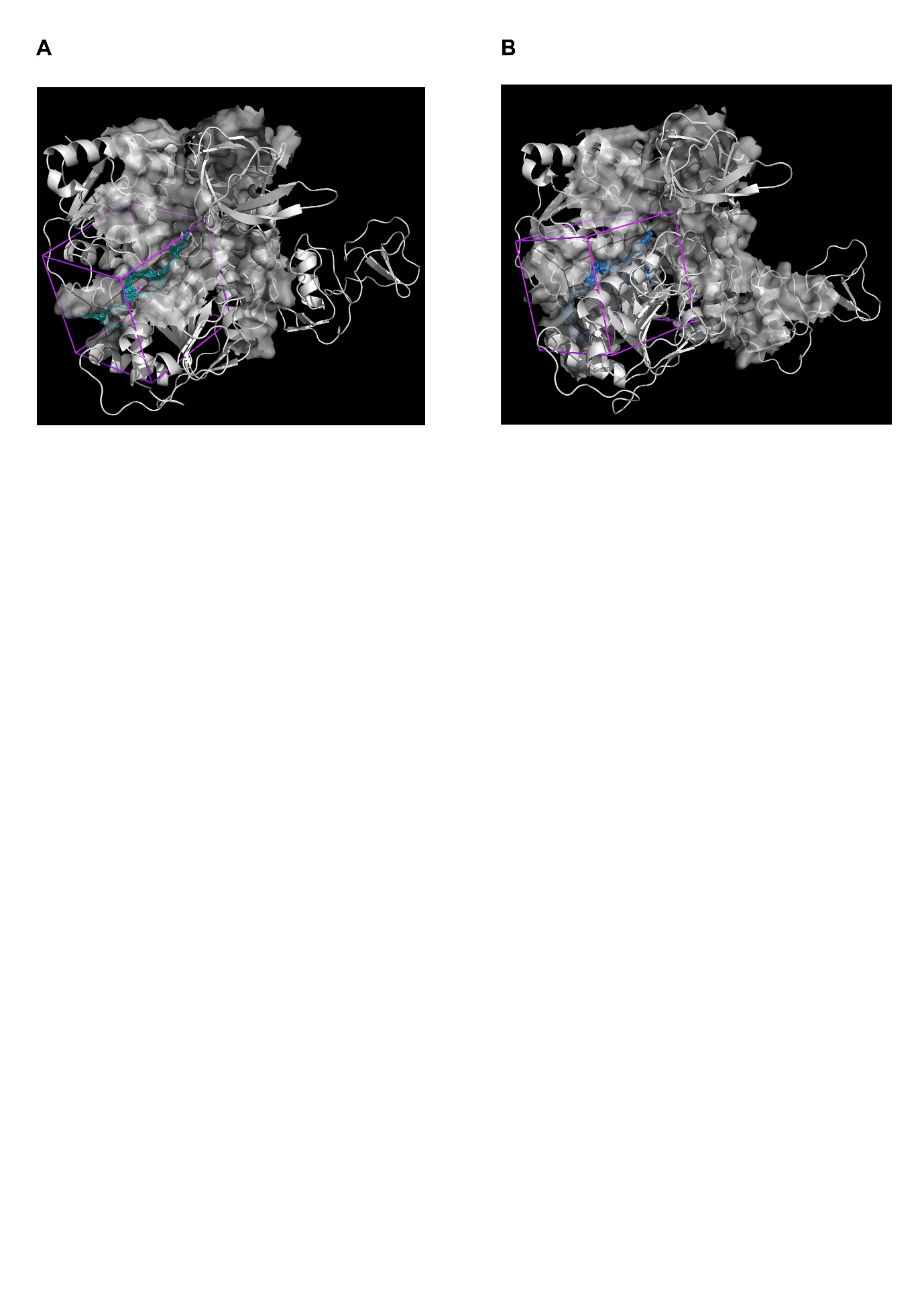


**Figure S4. Predicted binding sites of ATP and ATPγS on SARS-CoV-2 nsP13.** Binding models were generated using AMDock molecular docking software. (A) Predicted binding conformation of ATP within the nucleotide-binding pocket of nsP13. (B) Predicted binding conformation of the non-hydrolyzable analog ATPγS at the same site. Ligand interactions were visualized and analyzed to identify key residues involved in nucleotide recognition and binding. These models provide structural context for the biochemical differences observed in nsP13 function in the presence of ATP versus ATPγS.

**Supplementary Fig. S5**


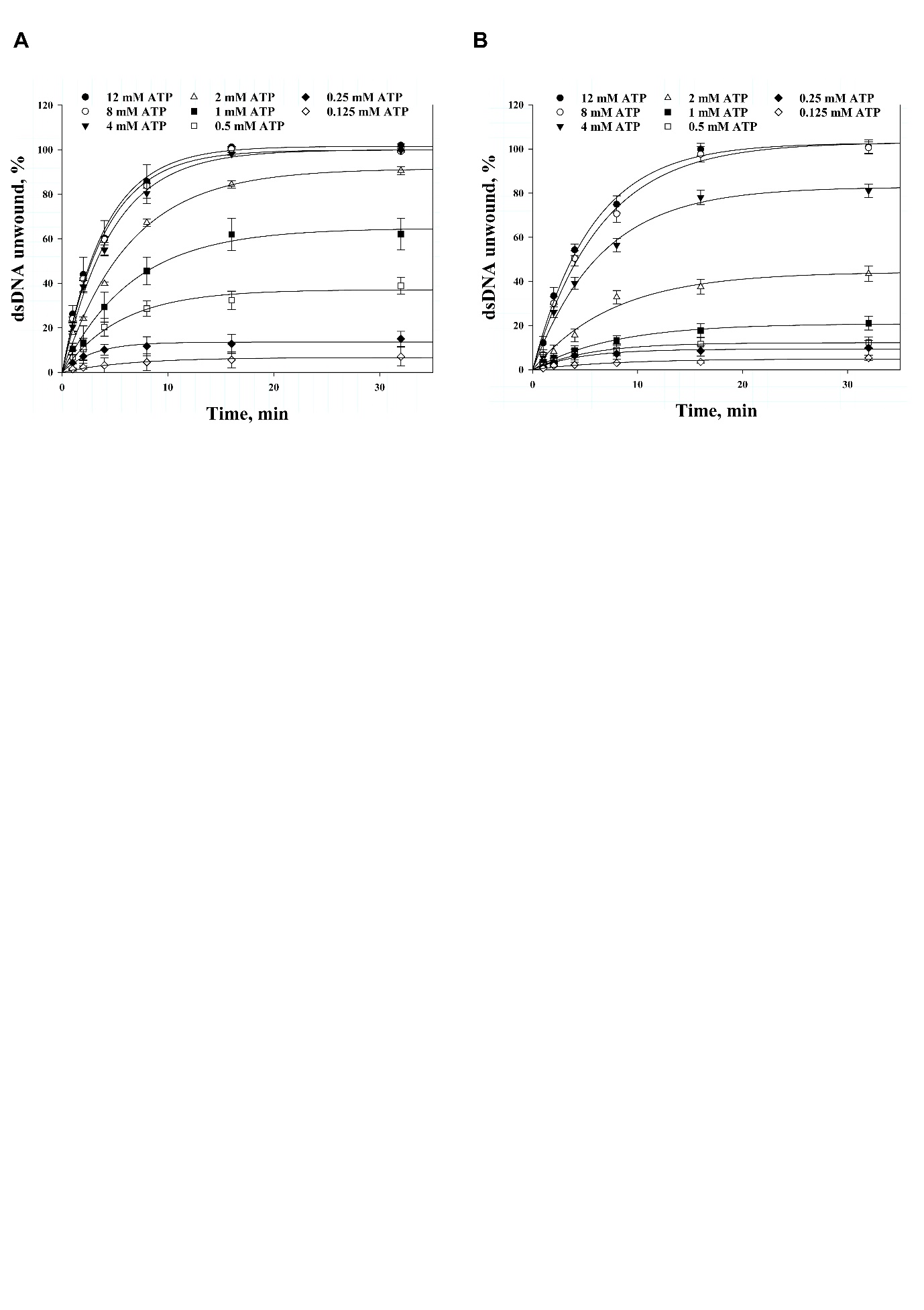


**Figure S5. Inhibition of nsP13-catalyzed dsDNA unwinding by ATPγS.** (A) Unwinding activity of nsP13 as a function of ATP concentration in the presence of a constant Mg²⁺ concentration. Reactions were performed at 37°C using various concentrations of ATP as indicated. The resulting amplitudes of dsDNA unwinding were: ● (12 mM ATP), 100%; ○ (8 mM ATP), 100%; ▼ (4 mM ATP), 100%; ∆ (2 mM ATP), 91%; ■ (1 mM ATP), 64%; □ (0.5 mM ATP), 37%; ♦ (0.25 mM ATP), 13%; ◊ (0.125 mM ATP), 6.6%. (B) Unwinding activity of nsP13 in the presence of a fixed concentration of ATPγS (0.6 mM) and varying ATP concentrations. Reactions were conducted under the same conditions as in panel A. The resulting amplitudes were: ● (12 mM ATP), 100%; ○ (8 mM ATP), 100%; ▼ (4 mM ATP), 82%; ∆ (2 mM ATP), 44%; ■ (1 mM ATP), 20%; □ (0.5 mM ATP), 12%; ♦ (0.25 mM ATP), 9.3%; ◊ (0.125 mM ATP), 4.8%. All data represent the mean values from independently repeated experiments, with error bars indicating the standard deviation. These results demonstrate a dose-dependent inhibition of dsDNA unwinding by ATPγS, particularly under limiting ATP conditions.

**Supplementary Fig. S6**


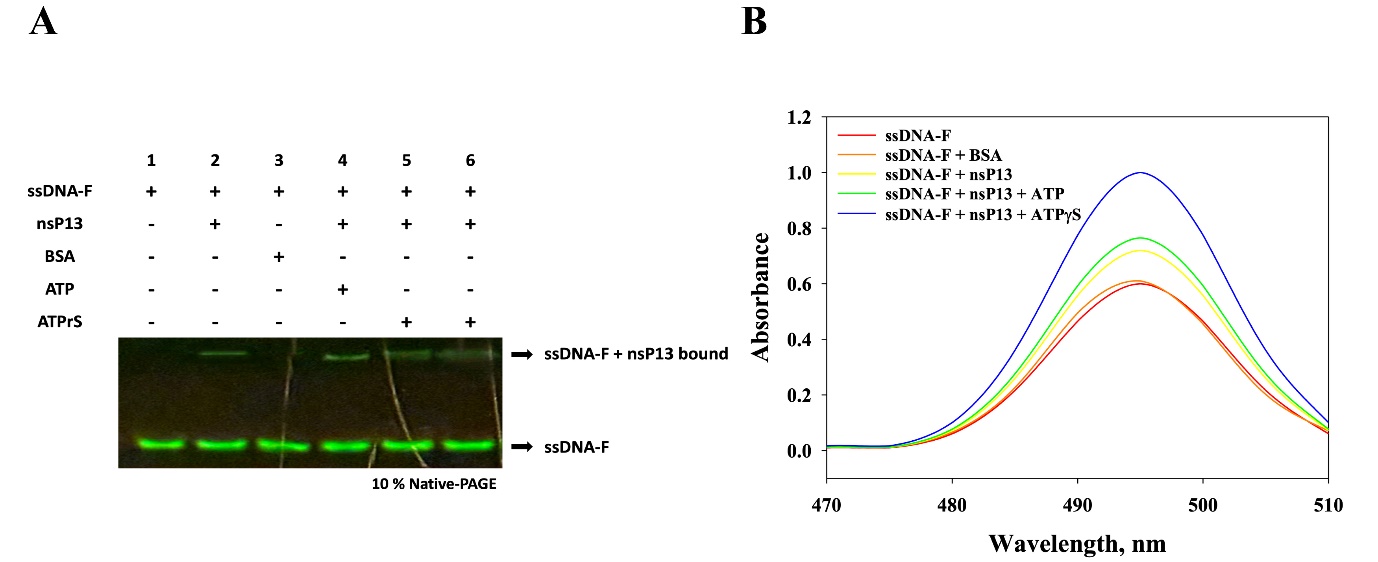


**Figure S6. Fluorescence enhancement of fluorescein-labeled ssDNA upon binding to nsP13.** (A) Electrophoretic mobility shift assay (EMSA) assessing the binding of nsP13 to 5′-fluorescein-labeled 25-mer ssDNA (ssDNA-F, 500 nM) under six different conditions. Samples were resolved on a 10% native PAGE gel and visualized using UV/Vis imaging after 30 minutes of electrophoresis. Lane contents (left to right): ssDNA-F alone; ssDNA-F + nsP13; ssDNA-F + BSA; ssDNA-F + nsP13 + ATP; ssDNA-F + nsP13 + 30 nM ATPγS; ssDNA-F + nsP13 + 60 nM ATPγS. (B) Absorbance spectra of the same samples shown in (A), recorded from 470 to 510 nm to evaluate changes in fluorescein absorbance upon protein binding. Traces are color-coded: red, ssDNA-F alone; orange, ssDNA-F + BSA; yellow, ssDNA-F + nsP13; green, ssDNA-F + nsP13 + ATP; blue, ssDNA-F + nsP13 + ATPγS.

**Supplementary Fig. S7**

**Figure S7. Fluorescence anisotropy of nsP13 binding to 25-nt fluorescein-labeled ssDNA-F at high concentration.** Fluorescence-based binding assays were performed to assess the interaction between nsP13 and 5′-fluorescein-labeled 25-mer ssDNA (ssDNA-F) under various nucleotide conditions. Reactions were conducted at 25 °C for 5 minutes using 5.0 μM ssDNA-F—10-fold higher than the DNA concentration used in Figure 5A—and increasing concentrations of nsP13. Binding curves were generated in the presence of no nucleotide (●), ATPγS (○), or ATP (▼), and fitted to Equation (2). The resulting amplitudes and apparent K₁/₂ values were as follows: No nucleotide: 23,600 a.u.; K₁/₂ = 53 nM, ATPγS: 58,300 a.u.; K₁/₂ = 41 nM; ATP: 30,800 a.u.; K₁/₂ = 34 nM.

**Supplementary Fig. S8**

**Figure S8. Stoichiometric binding of SARS-CoV-2 nsP13 to 25-mer ssDNA under different nucleotide conditions.** Fluorescence-based binding assay measuring the interaction between nsP13 and 5′-fluorescein-labeled 25-mer ssDNA (ssDNA-F) under varying nucleotide conditions. Reactions were conducted at 25 °C for 5 minutes using a fixed concentration of ssDNA-F (50 nM) and increasing concentrations of nsP13. Binding curves were generated in the presence of ATPγS (●), ATP (○), or no nucleotide (▼). The fitted amplitudes and apparent K₁/₂ values were as follows: ATPγS, 11,300 a.u. and 35 nM; ATP, 8,200 a.u. and 40 nM; no nucleotide, 6,500 a.u. and 43 nM. These results indicate nucleotide-dependent modulation of ssDNA binding affinity by nsP13.

**Supplementary Tables**

**Table S1. Sequences of the single-stranded oligonucleotides used in this study.**

| **Name** | **Structure** | **DNA sequences of top strand *** |
| --- | --- | --- |
| **20T25bp** | 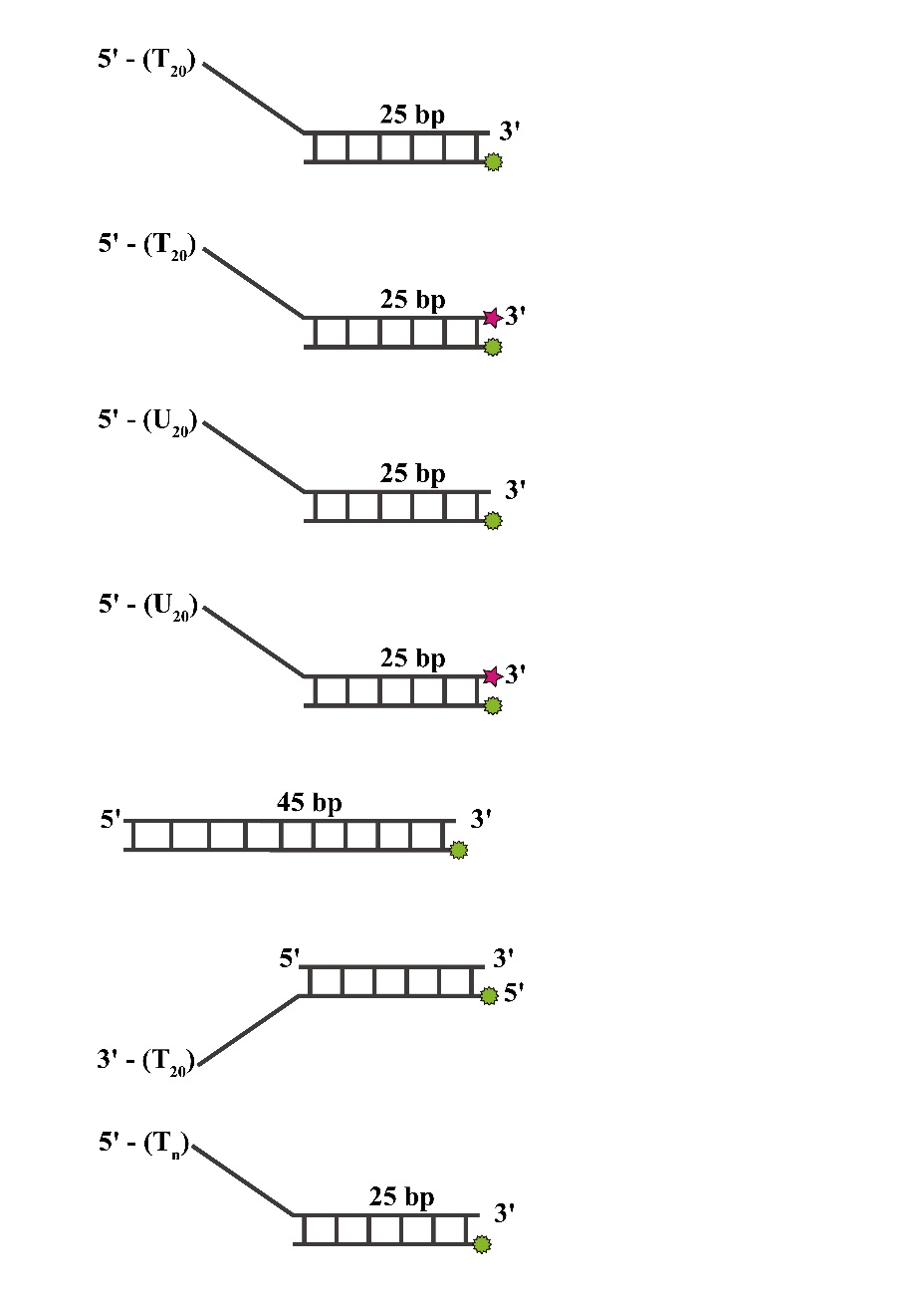 | **5'–T20–GAGCGGATTACTATACTACATTAGA–3'** |
| **20T25bp-T** |  | **5'–T20–GAGCGGATTACTATACTACATTAGA– (TAMRA)–3'** |
| **20U25bp** |  | **5'–U20–GAGCGGAUUACUAUACUACAUUAGA–3'** |
| **20U25bp-T** |  | **5'–U20–GAGCGGAUUACUAUACUACAUUAGA– (TAMRA)-3'** |
| **45bp** |  | **5'–T20–GAGCGGATTACTATACTACATTAGA–3'** |
| **3' 20T25bp** |  | **5'–GAGCGGATTACTATACTACATTAG–3'** |
| **T_n_25bp** |  | **5'–T(5, 6, 7, 8, 9, 10, 11, 12, 13, 14, 15)–GAGCGGATTACTATACTACATTAGA–3'** |

*Underlined sequence denotes the bottom strand.
